# Focus on Performance: The 21^st^ Century Revolution in Medical Education

**DOI:** 10.4103/0973-1229.37085

**Published:** 2008

**Authors:** Frank Davidoff

**Affiliations:** * *Editor Emeritus, Annals of Internal Medicine and Executive Editor, Institute for Healthcare Improvement*

**Keywords:** Competence, Experiential learning, Improvement, Medical education, Performance

## Abstract

For centuries medicine was predominantly a tradition-based “trade” until the introduction of science transformed it into an intellectually rigorous discipline. That transformation contributed heavily to the dominance in medical education of the learning of biomedical concepts (“knowing that”) over learning how to translate that knowledge into clinical performance (“knowing how”). The recent emergence of performance-oriented educational initiatives suggests, however, that the balance between these two complementary approaches is changing, a change that has been referred to as “the Flexnerian revolution of the 21^st^ century.” Problem-based learning, learning the practice of evidence-based medicine, and learning to use clinical guidelines are among the important initiatives designed to develop high-level performance in the care of individual patients. Initiatives in which learners acquire skill in changing the performance of care systems are also being widely implemented. These trends have received important formal support through recent changes in residency training accreditation standards. Although it is too early to assess the impact of these initiatives or to know whether they will develop further, medical education is unlikely to reach its full potential unless it successfully comes to grips with the challenges of understanding, teaching, and measuring performance.

## Introduction

Medicine has always been a “learned” profession, and doctors are often referred to as “learned intermediaries;” that is, they are understood to be the ultimate brokers between knowledge and practice in the domains of illness and health. Both the practice of medicine and medical education are therefore “hybrids,” involving both conceptual and working knowledge.

But despite frequent reaffirmation of their mantra of “knowledge, skills, and attitudes,” medical educators have during the past 150 years focused their attention, energy, and resources predominantly on the first of these: the “transcription” of conceptual biomedical knowledge into the heads of medical students and residents. Once that transcription is accomplished, medical education is less clear about how students should learn to “translate” their hard-earned biomedical knowledge into reliable, safe, and efficient clinical performance (Armstrong, Mackey and Spear, 2004). Indeed, recent documentation of the glaring deficiencies in the quality and safety of care delivery have demonstrated convincingly that this translation process is far from ideal (Institute of Medicine, 2001), leaving little doubt that there exists a serious “knowledge-performance” gap.

Fortunately, medical education in the US has begun taking the translation process more seriously than it had in previous years. No longer is it sufficient for medical students and residents to “know that” certain generalizable biomedical principles and facts are true; they are now expected to “know how” to apply that knowledge in individual patients and groups of patients in ever more sophisticated ways. This increase in expectations regarding working knowledge is reassuring, since it reflects what is known about the process by which learners become true professionals: moving from being a novice through the stages of advanced beginner, acquiring competence and proficiency and, ultimately, achieving high-level expertise (Dreyfus and Dreyfus, 2000). More specifically, these expectations are reflected in a current and ongoing shift from a medical education curriculum focused mainly on structure and process to one concerned mainly with the development and measurement of competent performance – that is, the ability to act, to deliver care – a shift that has been referred to as “the Flexnerian revolution of the 21^st^ century” (Carracio *et al*., 2002). (The shift of medical education from an intellectually doubtful and largely commercial enterprise to a scholarly, university-associated discipline that was catalyzed by Flexner's devastating 1910 report on the state of medical education in the US (Flexner, 1910) is considered the original, 20^th^ century Flexnerian revolution.)

## Medicine: A Scholarly Discipline or a Trade?

There are many reasons why, in medical education, the transfer of conceptual biomedical knowledge has until recently overshadowed the development of competence. To begin with, it was, of course, the introduction of the scientific method that transformed medicine from an authoritarian, tradition-based trade to an authoritative, intellectually rigorous discipline, and conceptual knowledge is both instrument and product in science: explicit, abstract, precise, quantitative. Competence, in contrast, is like “dark matter” in astronomy: although it makes up most of the universe of working knowledge, we understand relatively little about it. What does it really consist of? Which of its components are most important? How do people acquire it? What's the best way to measure it? And how can you tell when they have enough of it? (Carracio *et al*., 2002; Epstein and Hundert, 2002).

But in addition to these cognitive and technical reasons for the imbalance between “learning that” and “learning how,” important social and emotional reasons have been at work in medical education as well. Pure or basic science is regarded in the scientific and scholarly communities as having greater explanatory power, hence greater intellectual worth, than applied or practical science. The social status of applied or “soft” sciences is therefore generally lower than that of pure or “hard” sciences (Hagstrom, 1975).

Fortunately, as we have noted elsewhere (Batalden and Davidoff, 2007a), there has been some recent progress in understanding the nature of working knowledge. Most importantly, it is increasingly clear that competence is acquired primarily through experiential learning – a four-element cycle (or spiral) in which learners move from direct personal involvement in experiences, to reflection on those experiences, integration of their observations with sense-making concepts and mental models, and finally back to more experiences. Formal training for all high-performance (applied) professions, for example, music, architecture, theater, and athletics, is grounded in the unique requirements of experiential learning: case-based coaching, rather than lectures by content experts; hands-on, practicum experiences (including simulations, if necessary) in addition to written end-objectives; repeated experiences and outcome evaluations over time rather than initial, one-shot exercises; and, ultimately, acquisition of the advanced skills of “reflection-in-action,” which is required for high-level performance and “reflection-on-action,” which is required for continued self-evaluation and self-instruction (Schon, 1987).

The delivery of medical care is a form of performance; in fact, medicine is arguably an extremely high-performance profession. As late as the mid-nineteenth century, however, medical education included only fragments of the experiential learning cycle – minimal classroom exposure to (inadequate) biological and clinical concepts, without linkage to actual case understanding and management; and hit-or-miss apprenticeships, with little connection of cases to meaningful conceptual knowledge. In the US, experiential learning moved into the medical education mainstream in a meaningful sense only in the late 1800s, primarily under the influence of Osler's innovation of hospital-based clinical clerkships (Ludmerer, 1985).

Despite the introduction of clerkships into the formal structure of medical education, followed soon thereafter by residency and fellowship training, the primary focus of medical educators has been, and remains, on cognition – helping learners understand the biomedical concepts that are medicine's unique intellectual asset – rather than on competence – the working knowledge that determines the quality and consistency of performance (Carracio *et al*., 2002; Papa and Harasym, 1999). As a consequence, the experiential learning component of medical education continues to exist in uneasy equilibrium with the learning of abstract biomedical concepts (Ludmerer, 1985; Ludmerer, 1999).

Thus, for over 100 years, medical students in their preclinical years have been expected to acquire enormous (and ever-increasing) amounts of conceptual biomedical knowledge, primarily through lectures and readings, supplemented by seminars and lab exercises, and driven by written exams. Not surprisingly, students initially try to understand and solve clinical problems in terms of those basic concepts. But during their clinical clerkships and residencies they switch over almost entirely to experiential learning as they begin to solve the complex problems of real, individual patients in real-world contexts. In order to do so, they need to acquire the concepts, vocabulary, and logic distilled directly from the collective experience of managing illness; this concrete, working knowledge differs fundamentally from the abstract biomedical knowledge acquired during their preclinical years (Elstein, Shulman and Sprafka, 1978; deBruin, Schmidt and Rikers, 2005).

## Competence-Oriented Medical Education

Although medical education is notoriously resistant to change, a number of innovative educational initiatives specifically designed to help students acquire working knowledge have now taken root and are beginning to spread. As we have noted elsewhere (Batalden and Davidoff, 2007a), these programmes are directed at two very different but complementary levels of medical performance: the delivery of care to individual patients by individual providers, and the overall operation of the systems in which that individual care takes place.

## Educational Initiatives Designed to Develop Competence in Individual Patient Care

Three relatively recent educational initiatives in particular are designed to build competence in the translation of generalizable scientific knowledge into the care of individual patients: problem-based learning, the practice of evidence-based medicine, and the development and use of clinical guidelines.

### 1. Problem-based Learning

Introduced in the 1960s, problem-based learning replaces traditional passive biomedical knowledge transfer (lectures, readings) with a quasi-experiential method of acquiring basic biomedical concepts (Wood, 2003). In this problem-based approach, written cases rather than living patients (hence “quasi-experiential”) serve as triggers for the definition of students’ learning goals and for independent self-directed learning, which is then refined in small group discussions under coaching by faculty. This discipline models the thinking and action-taking that arise from patients’ stories in actual clinical practice, including the skills of searching and critically appraising the biomedical literature.

Roughly 80% of US medical schools now provide at least some problem-based learning to their students. Students from problem-based learning schools perform well on standardized tests of knowledge (Kincade, 2005); there is evidence that they may be particularly proficient at making clinical diagnoses, retaining knowledge, integrating basic science concepts into clinical problems, and acquiring up-to-date clinical concepts following graduation (Nandi *et al.*, 2000). Problem-based learning at the residency level also improves self-directed learning behaviours and in-training test scores (Ozuah, Curtis, and Stein, 2001). Students who participate in problem-based learning's intensive group process demonstrate better interpersonal skills and psychosocial knowledge than their counterparts from traditional programmes (Kincade, 2005; Nandi *et al.*, 2000; Ozuah, Curtis, and Stein, 2001). They may therefore possess more of the specific skills of leadership management, and cooperation needed for executing changes in the current complex environment of practice.

### 2. Evidence-based Medicine

As it is currently defined, the practice of evidence-based medicine involves systematic and judicious application of the best available research evidence in the clinical care of individual patients and patient groups. Learning rigorous methods for formulating clear, answerable clinical questions, searching the medical literature, and critically appraising research studies are the working-knowledge cornerstones of this discipline. Translating relevant evidence into practice requires learning how to judge the relevance of research information to the problems of individual patients, and applying it to those patients in the context of local practices. Teaching the practice of evidence-based medicine at the undergraduate, graduate, and practitioner level has been shown to improve clinical knowledge, critical appraisal skills, the use of original studies to answer clinical questions, attitudes about the role of evidence, and clinical behaviour. It has proven to be most effective when it is integrated with “bedside” clinical teaching, in contradistinction to its use in classroom settings (Coomarasamy and Khan, 2004; Bradley *et al*., 2002).

Unfortunately, many pragmatic obstacles – the intimidating amount and complexity of published evidence; lack of confidence that answers to clinical questions are available; inadequate literature searching and appraisal skills; and most importantly, lack of time – interfere with the practice of evidence-based medicine in real-world clinical settings. As a consequence, most clinical questions that come up in daily practice still remain unanswered (Dawes and Sampson, 2003), a major factor in the “pressure drops in the pipeline from the generation of research evidence to its consistent application in clinical decision-making” (Scott, 2007). Learning to use the assistance of clinical librarians, pharmacists, and other “informationists” is therefore becoming an increasingly important, if underutilized, educational strategy in the practice of evidence-based medicine (Shearer, Seymour and Capitani, 2002).

### 3. Learning to use Clinical Guidelines

Valid, credible clinical guidelines are based on exhaustive review and critical appraisal of the medical literature. Evaluating the quality of clinical guidelines and applying them in practice can therefore serve as important proxies for direct retrieval and use of research information. For these reasons, learning about how best to use clinical guidelines is now considered a legitimate and important element in continuing medical education. Well constructed and precisely worded clinical guidelines help to adapt research evidence to individual patients since they take into account a range of patient characteristics, including comorbidities (Michie and Johnston, 2004). Clinical guidelines and protocols also help to apply evidence from research to specific patients through “forcing functions” such as algorithms, standard order sets, and flow sheets – all invitations to attend to the modification of process and habit. Despite many barriers to their validity, generalizability, and effectiveness, the use of clinical guidelines and protocols has been shown to be capable of making practice more reliable and more consistent with evidence from research (Grimshaw *et al*., 2004).

## Initiatives Designed to Develop Competence in System Change

A wide variety of initiatives now allow learners to acquire the “how to” knowledge they need to improve dysfunctional care systems. Some of these initiatives are broadly conceived and provide learners with know-how across the entire scope of improvement-related knowledge systems (Batalden and Davidoff, 2007b) as they grapple with real-world problems in health care delivery systems. Other initiatives are targeted much more narrowly, allowing learners to develop specific skills. All of these efforts are now supported by a substantial published literature in the rapidly evolving “science of improvement,” including a growing number of discipline-specific journals (for example, the *Joint Commission Journal on Quality and Patient Safety* and *Quality and Safety in Health Care*), textbooks, and monographs. Importantly, some highly effective experiential learning in these areas is taking place outside academic centers.

### 1. Initiatives that are Broad in Scope

In 2002 Gould and colleagues (Gould *et al*., 2002) reported on an innovative clinical clerkship for second-year medical students. Working in community-based practices, the students who participated in this clerkship were involved in designing and implementing a plan to improve the care of a population of diabetic patients. They first formulated a clear improvement aim for the target population, then characterized the patient group by collecting baseline data on over 500 patients. They designed and helped to implement an improvement intervention that fit in with the nature of the participating practices and worked to assure that the intended changes were put into practice. Follow-up measurements at six months revealed that the proportion of office visits with foot examinations had increased from 51% to 70%, the proportion with eye examinations had risen from 27% to 38%, and the patients’ mean glycosylated hemoglobin level had decreased from 7.7% to 7.2%, all statistically significant changes. In a formal assessment of the experience, the participating students acknowledged the benefits of outcomes management in clinical practice – as well as the tedium of medical records abstraction.

As in all traditional clinical clerkships, the students in this new clerkship were engaged in experiential learning – learning by doing – by taking care of real patients in real practices, and what they acquired in the process was working knowledge or know-how. What was different was that rather than simply learning how to deliver care, students were learning how to improve it – that is, to consciously and systematically bring the performance of a portion of the health care system closer to what the available scientific evidence says it can and should be (Institute of Medicine, 2001). Many similar broad, system-oriented initiatives in education have now been reported. A recent systematic review concluded that many of those initiatives have been effective in improving knowledge about quality improvement, attitudes toward health systems, and participation in quality improvement activities (Boonyasai *et al.,* 2007). The review also noted, however, that learners’ knowledge about improvement improved more than clinical outcomes, clinical benefits did not occur when learner behaviours did not change, and the use of adult learning principles did not improve educational outcomes. At the same time, the review did find that clinical outcomes were more likely to improve when the teaching methods were appropriate for experiential learning and included, for example, the provision of quality improvement tools, individualized coaching, and learners’ involvement in iterative tests of change (Batalden and Davidoff, 2007a).

### 2. Narrowly Focused Programmes

Other system-related improvement-oriented learning has been more narrowly focused. For example, teamwork training, based largely on the “crew resource management” training that has helped make aviation increasingly safe, has been introduced in a number of educational settings. Designed to develop high-level skills of both leadership and “followership,” these programmes appear to be particularly valuable for providers in areas (for example, surgery and emergency medicine) characterized by extreme complexity, major time pressure, rapidly changing information load, as well as high ambiguity, workload, and risk (Thomas, Sexton and Helmreich, 2004). Similarly, as part of the effort to prevent the failures in communication that account for a large proportion of adverse patient events, many providers are being trained in the use of structured communication tools such as the SBAR (situation-background-assessment-recommendation), situation briefing model, and other related techniques (Leonard, Graham and Bonacum, 2004).

## Factors Affecting the Development of Performance-Oriented Education Initiatives

Successful development of any educational initiative depends at least in part on the ability to assess its impact. Although the assessment of professional competence and performance continues to present difficult challenges (Carracio *et al*., 2002; Epstein and Hundert, 2002), a variety of instruments are now available that provide reasonably reliable process measures of individual clinical performance (Durning *et al*., 2002; Peabody, Luck and Glassman, 2004; Branch, 2005; Toolbox of assessment methods, 2005). Learning portfolios – collections of materials made by learners that record key elements in their training and careers – appear to be uniquely suitable for reflective observation on performance (Challis, 1999). An example of particular interest is the PCDiary, a national electronic learning portfolio into which nearly all Canadian physicians now regularly enter “learning items” (Dornan, Carroll and Parboosingh, 2002). It allows physicians to compare their learning needs and practices with those of their peers. Analysis of aggregated data from PCDiary has revealed that reading the medical literature is itself the most frequent stimulus for further learning. Reviewing the management of more than one patient emerges as the strongest determinant of a commitment to make a change in practice (Campbell *et al*., 1999).

Momentum generated by the growing experience with improvement efforts, and the increasing effectiveness of those efforts, will help to drive expansion of performance-oriented learning programmes at all levels of medical education. Perhaps more important, these programmes are no longer optional in the US. The Accreditation Council for Graduate Medical Education (ACGME) now includes “practice-based learning and improvement” (which focuses on individual patient care) and “systems-based practice” (which focuses on care systems) among the six competencies that residents in all specialties are expected to acquire (Batalden *et al*., 2002). Programme directors throughout the USA are actively developing learning experiences designed specifically for that purpose. The 24 members of the American Board of Medical Specialties (ABMS) have recently adopted Maintenance of Certification requirements built on the same six competencies and will require evidence from each certified mid-career physician that they are being exercised (American Board of Medical Specialties, Evanston, IL., 2006).

Of course, only time will tell whether performance-oriented learning initiatives really represent a major and enduring shift in medical education or will prove to be yet another in the long history of failed educational reforms (Carracio *et al*., 2002; Ludmerer, 1985; Papa and Harasym, 1999). It is already clear, however, that their future development faces many obstacles: the prevailing push of clinicians toward piece-work productivity diminishes the time available for reflection; individualized coaching is more expensive and time-consuming for faculty than lecturing, and relatively few faculty have been trained to be expert coaches; and confronting performance shortcomings can be painful. Moreover, medical faculty generally have little experience in rigorously evaluating the clinical performance of the systems in which they work; it is hard to build evidence-based medicine into teaching unless it has been solidly incorporated into the faculty's own clinical practices (Richardson, 2005); and medicine's autonomy-driven culture can make it difficult for both teachers and learners to adopt the kind of shared decision-making required in high reliability organizations.

## The Implications of Performance-Oriented Learning Initiatives for Medicine and Medical Education

Successful improvement in performance at both the individual and system level will demand continuing discoveries of new ways to understand and influence the complex social systems in which improvement takes place (Batalden and Davidoff, 2007a; Boonyasai *et al*., 2007), as well as a progressive increase in understanding the nature of experiential learning (Schon, 1987). At the same time, these challenges create important research and academic opportunities for medical faculty which, over time, can help to legitimize the epistemology of improvement in academic settings. Those opportunities may, in turn, enable improvement science to command new sources of funding, venues for publication, and criteria for academic promotion, thus strengthening the teaching infrastructure and catalyzing a further evolution of the role of experiential learning in medicine.

These changes need not dilute or diminish the importance of the traditional scientific base or of conceptual learning in medicine and medical education. On the contrary, since generalizable scientific evidence is an essential element of improvement science, strengthening improvement-oriented experiential learning can only enhance the value of that evidence by linking it more directly and effectively with the process of health care delivery.

## Concluding Remarks

Given the enormous complexity and inertia of the health care system, meaningful improvement in health care is unlikely to be sustained unless improvement efforts within the care system itself are linked to concurrent and equally serious efforts to foster better professional development. Indeed, everyone involved in health care will need to understand that the experiential learning involved in improving their work is as much part of their job as doing that work (Batalden and Davidoff, 2007a). Sophisticated practice-based, performance-oriented learning programmes will therefore be increasingly needed if medicine is to continue meeting one of its most fundamental professional obligations – an obligation of all professions – namely, unceasing movement toward new levels of performance (Batalden and Davidoff, 2007b).

### Take Home Message

For over 100 years medical education has focused primarily on the transfer of conceptual biomedical knowledge, rather than on acquisition of “know how” – the working knowledge that learners need in order to translate that knowledge into performance: that is, consistent delivery of rational, effective, and safe clinical care. In the past two decades, performance-oriented educational innovations have made their appearance, but in order to succeed they will need strong support and close ties to the efforts being made to improve both clinical processes and outcomes.

## Questions That This Paper Raises

1. What factors have led to the dominance of conceptual knowledge transfer in medical education?
2. What are the essential elements of experiential learning?
3. Is there convincing evidence that performance-oriented educational initiatives have been effective in improving care delivery?
4. What are the barriers to further development of performance-oriented programmes in medical education?
5. How can performance-oriented medical education programmes be integrated with efforts to improve clinical processes and outcomes?

## About the Author

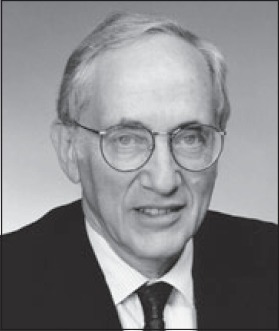



Frank Davidoff, MD, MACP is an internist who served on the faculty at Harvard University and University of Connecticut medical schools prior to becoming senior vice-president for education at the American College of Physicians and then editor of Annals of Internal Medicine. He is now emeritus editor of Annals of Internal Medicine and executive editor for the Institute for Healthcare Improvement. He served as a member of the Non-Prescription Drug Advisory Committee of the Food and Drug Administration, and is vice-President of Physicians for Human Rights. His publications include more than 130 original papers, editorials, and chapters, and a book of essays, “Who Has Seen a Blood Sugar? Reflections on Medical Education.”
